# Can red yeast rice and olive extract improve lipid profile and cardiovascular risk in metabolic syndrome?: a double blind, placebo controlled randomized trial

**DOI:** 10.1186/s12906-015-0576-9

**Published:** 2015-03-10

**Authors:** Veronique Verhoeven, Anastasia Van der Auwera, Luc Van Gaal, Roy Remmen, Sandra Apers, Michel Stalpaert, Johan Wens, Nina Hermans

**Affiliations:** Academic center for Primary and Interdisciplinary care, Faculty of Medicine and Health Sciences, University of Antwerp, Antwerp, Belgium; Natural Products and Food Research & Analysis (NatuRA), Department of Pharmaceutical Sciences, University of Antwerp, Universiteitsplein 1, Antwerp, Belgium; Department of Endocrinology, Diabetology, and Metabolism, Faculty of Medicine, Antwerp University Hospital, 2650 Edegem, Antwerp, Belgium; Laboratory of Molecular and Clinical Pathology (RIATOL), AML, Sonic Healthcare Benelux, Emiel Vloorsstraat 9, Antwerp, 2020 Belgium

**Keywords:** Cardiovascular prevention, Metabolic syndrome, Statins, Red yeast rice, Olive, Cholesterol

## Abstract

**Background:**

Metabolic syndrome (MetS) comprises a spectrum of clinical phenotypes in which dyslipidemia, dysglycemia and hypertension are clustered and where all share a high level of oxidative stress and an increased risk of cardiovascular disease. This study examines the effect of a nutritional supplement combining red yeast rice and olive fruit extract on the lipid profile and on oxidative stress in a population of patients with MetS.

**Methods:**

In a double blind placebo controlled randomized trial, 50 persons with MetS, as defined by the ATPIII criteria, received the study product or placebo for 8 weeks. The study product contained 10.82 mg of monacolins and 9,32 mg of hydroxytyrosol per capsule, and is commercialized as Cholesfytol plus. The primary outcome measure was the difference in LDL reduction between intervention and control groups. Furthermore, differences in changes of CH, HDL, ApoA1, ApoB, HbA1c and oxLDL were measured, as well as side-effects, CK elevation, changes in clinical parameters and in cardiovascular risk.

**Results:**

In the intervention group, LDL cholesterol was lowered by 24% whereas it increased by 1% in the control group (p < 0.001). Other effects observed were a change in total cholesterol (−17% in the intervention group vs +2% in the control group, p < 0.001), apolipoprotein B (−15% vs +6%, p < 0.001), and TG (−9% vs + 16%, p = 0.02). Oxidized LDL decreased by 20% vs an increase of 5% in the control group (p < 0.001). Systolic and diastolic arterial blood pressure decreased significantly by 10 mmHg (vs 0% in the control group, p = 0.001) and 7 mmHg (vs 0% in the control group, p = 0.05) respectively. One person in the intervention group, who suffered from Segawa’s syndrome, dropped out because of severe muscle ache.

**Conclusions:**

The combination of active products in this study may be an alternative approach to statins in people who do not need, or cannot or do not want to be treated with chemical statins. Side effects, effects on oxidative stress and on glucose metabolism need to be examined more thoroughly.

**Trial registration:**

Clinicaltrials.gov NCT02065180 (February 2014).

## Background

With one death in the US every 40 seconds, cardiovascular disease (CVD) remains the leading cause of mortality in the industrialized world [1]. Whereas medical treatment of risk factors, especially hypertension and dyslipidemia, has become more strict and evidence-based, the prevalence of unfavorable metabolic conditions such as obesity and diabetes in the population is increasing.

Depending on the definition used, 30 to 40% of Americans suffer from metabolic syndrome (MetS) [2]; in developing countries this trend is rising as well [3] and the worldwide prevalence is estimated to be around 25% [4]. Metabolic syndrome comprises a broad clinical spectrum and the definition and concept are matter of debate; however, the clustering of dyslipidemia, dysglycemia, intra-abdominal fat accumulation and hypertension unequivocally leads to an increased risk of developing CVD and diabetes [5].

Management of the MetS is essentially multifaceted [6]; the cornerstone is change of lifestyle, and medical treatment includes antihypertensive therapy and treatment of dyslipidemia for subjects at greater risk [7]. Whereas statin treatment effectively reduces CVD in secondary prevention and is standard therapy for primary prevention in diabetes, there is no consensus in favor of a generalized use for primary prevention in the heterogeneous group of patients with MetS.

So called functional foods and nutritional supplements may be an attractive alternative for people who are reluctant to take drugs. In this context, red yeast rice (RYR) supplements are becoming increasingly popular. These over-the-counter products have been shown to have a lipid lowering effect in an increasing number of clinical studies. In 2011, the European Food Safety Authority (EFSA) affirmed a health claim of a cause and effect relationship between daily consumption of 10 mg of monacolin K from fermented red yeast rice preparations and maintenance of normal blood LDL cholesterol concentrations [8].

Olive oil, more specifically polyphenols in olive extract, may have some anti-oxidative properties and is therefore another candidate food supplement in MetS. Oxidative stress plays a major role in metabolic syndrome-related clinical manifestations [9]. EFSA states that daily consumption of 5 mg of olive oil polyphenols (standardised by the content of hydroxytyrosol and its derivatives) protects LDL particles from oxidative damage [10]. Other health claims related to olive oil, such as maintenance of normal blood HDL-cholesterol concentrations or maintenance of normal blood pressure, have been rejected by EFSA based on a lack of existing evidence.

These two extracts have not been tested together before and have not been tested in people with the specific metabolic dysregulation as seen in metabolic syndrome. In this study, we examine the effects of a commercially available red yeast rice and olive extract food supplement on lipid status and oxidative stress estimates in a population of patients with metabolic syndrome.

## Methods

We conducted a double blind, placebo controlled, randomized controlled trial in 50 patients from mid-February to mid-April 2014. Eligible participants were patients suffering from metabolic syndrome as defined by the ATPIII criteria [11]. People with metabolic syndrome should meet three or more of these criteria (Table 1).

**Table 1:** **ATPIII criteria for diagnosis of metabolic syndrome**

**Risk factor**	**Defining level**
Abdominal obesity, given as waist circumference	
Men	>102 cm
Women	>88 cm
Trigiycerides	> = 150 mg/dL
HDL cholesterol	
Men	<40 mg/dL
Women	<50 mg/dL
Blood pressure	> = 130/> = 85 mm Hg or drug treatment for hypertension
Fasting glucose	> = 110 mg/dL

Exclusion criteria were: age <18 years, treatment with any cholesterol lowering drugs, chronic inflammatory diseases, known type 2 diabetes, fasting triglyceride level > 400 mg/dL [12], or desire to become pregnant during the study period.

Factors possibly having an effect on lipid levels and/or oxidative stress were registered by means of a questionnaire, which included a detailed registration of dietary habits, physical exercise, smoking habits, menopausal status, and perceived level of stress in daily life. A cardiovascular risk profile was determined by means of the SCORE table [13], calibrated for Belgium. Candidates taking cholesterol lowering food products (for example drinks or margarines enriched with plant sterols) needed to stop these products prior to the study (wash out period of 2 weeks).

A sample size calculation based on SD (standard deviation) values in previous studies, determined that 40 patients were needed to establish a difference in LDL-cholesterol reduction of 15% between the intervention and control groups (power 0.80, significance level 0.05). LDL difference was chosen because it is the primary target of statins [14].

Participants were randomized to the control or intervention group by random generation of even (intervention) and uneven (control) numbers by means of a computer program. No stratification for age, sex or cholesterol level was performed. Study groups were blinded to participants as well as researchers. Written informed consent was obtained from each participant at the time of inclusion.

We used a commercially available food supplement and a placebo which looked similar to the original product. The content of the capsules was analysed by an independent laboratory, certified by the Belgian Federal Public Service for health, food chain safety and environment (University Centre for Analysis of Pharmaceuticals and Health products). The content of monacolins (sum of Monacoline K and Lovastatin) was determined using an optimized HPLC-UV method, based on the analytical protocol described by Yong-Guo et al. [15], and compared with lovastatin (CRS, EDQM Strasbourg France) as an external standard. For the determination of hydroxytyrosol, a suitable aliquot of the supplement was sonicated in 80 mL of mobile phase for 15 min. After cooling to room temperature the sample solution was topped up to 100 mL using the same solvent. The test and reference solution (10.0 mg hydroxytyrosol (purity 100%, Extrasynthese, France)/50.0 mL mobile phase) were analyzed by means of HPLC at a wavelength of 233 nm. The analysis was performed on a reversed phase C18 column using an isocratic mixture of trifluoroacetic acid 0.15% in water and methanol 80:20 as mobile phase. All analyses were performed in triplicate on two different days (n = 6). Each capsule contained 10.82 +/− 0.84 mg of monacolins (of which 5.88 +/−0.46 mg of lovastatin), and 9.32 +/− 00.54 mg of hydroxytyrosol. The study product and placebo were administered in a neutral, white container. Study participants were instructed to take 1 capsule every evening for 8 weeks. No specific dietary measures were imposed.

Clinical examination was performed at baseline and at the end of the study, and included: measurement of weight, height and waist circumference (measured at the midpoint between the lowest rib and the iliac crest); and determining blood pressure by means of an aneroid sphygmomanometer. All measurements were performed by the same clinician.

Blood sampling was performed at the beginning and at the end of the study period: fasting plasma cholesterol (CH), HDL, LDL, triglycerides, apolipoprotein A1 (apoA1), apolipoprotein B (apoB), fasting glucose and HbA1c were measured. Furthermore, we measured oxidized LDL (oxLDL); OxLDL represents a mixture of oxidatively modified LDL particles, containing oxidatively modified lipid and apoB protein components. Although an indirect biomarker of oxidative stress, assessment of the oxidative modification of LDL is relevant given its pro-atherogenic properties [16-18]. Additionally, creatine kinase (CK) was measured as a parameter linked to side-effects. Glucose, being an inclusion criterion, was only measured at baseline. As in most clinical trials and in clinical practice, the Friedewald formula [19] was chosen to calculate LDL concentrations. Analyses were performed in a certified commercial clinical laboratory (Laboratory of Molecular and Clinical Pathology (RIATOL), AML, Sonic Healthcare Benelux, Emiel Vloorsstraat 9, 2020 Antwerp, Belgium). In accordance with routine practice, CH, HDL, TG, CK and glucose were measured by means of spectrophotometry; apoA1 and apoB by means of nephelometry; and HbA1c by means of high pressure liquid chromatography. Plasma oxLDL was analyzed by an enzyme immunoassay kit (Mercodia OxLDL sandwich ELISA kit, Mercodia AB, Uppsala, Sweden). Mercodia OxLDL ELISA is a solid phase two-site enzyme immunoassay, in which two monoclonal antibodies are directed against separate antigenic determinants on the oxidized apolipoprotein B molecule. OxLDL in the plasma sample reacts with anti-oxidized LDL antibodies bound to microtitration wells (mAb 4E6) and horse radish peroxidase (HRP) conjugated anti-oxidized LDL antibodies in the solution. The oxidation of 3,3’,5,5’-tetramethylbenzidine (TMB) by HRP is measured spectrophotometrically at 450 nm.

At the end of the study, participants were asked to complete a structured questionnaire on compliance, dietary or lifestyle changes, and side-effects. They were specifically asked for the following possible side-effects: muscle ache, muscle weakness, muscle cramps, arthralgia, sleeping disorders, depression and hair loss [20]. Furthermore, participants could report other possible side-effects. Visual analogue scales were used to quantify the severity of side-effects.

The primary outcome measure is the difference in LDL reduction between both groups. Secondary outcome measures are differences in reduction of other lipids (total CH, HDL, TG) and of apoA1, apoB, HbA1c and oxLDL. Furthermore we analyzed side effects, CK elevation, clinical parameter changes and cardiovascular risk changes.

Statistical analyses were performed using SPSS, SPSS Inc, Chicago, Illinois, USA. We used an independent samples *t*-test to compare the effect of the intervention in both groups. As an exploratory analysis, we performed multivariate linear regression to examine effect modification within the intervention group.

The study protocol was approved by the ethical review board of the Antwerp University Hospital, and it was registered on 13/02/2014 at clinicaltrials.gov, nr NCT02065180.

## Results

Fifty participants who met the inclusion criteria entered the study and were randomly allocated to the intervention group (n = 26) or the control group (n = 24). Participants met three (n = 43) or four (n = 7) of the ATPIII criteria. Most people had an elevated blood pressure (44/50) and/or a large waist circumference (35/50). Twenty participants had an increased triglyceride level, 19 a decreased HDL level, 13 were on antihypertensive medication and four had (previously unknown) elevated fasting glucose.

**Figure 1:**
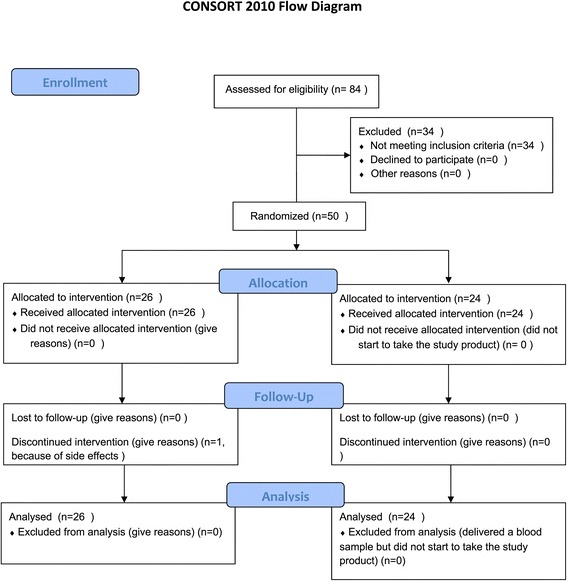
**Flow of patients in the study.**

Baseline characteristics are shown in Table 2. Intervention and control groups were similar except for mean diastolic blood pressure, which was higher in the control group. The flow of patients in the study is shown in Figure 1 (end of manuscript).

**Table 2:** **Baseline characteristics of study participants**

	**Intervention (n = 26)**	**Control (n = 24)**	p-value*
**Mean age (SD)**	**53.6 (8.4)**	**49.9 (13.3)**	**0.25**
Sex	Female: 17	Female: 13	0.42
	Male: 9	Male: 11	
Waist circumference (cm)	96.8 (10.2)	96.7 (10.4)	0.98
Body mass index (kg/m^2^)	27.8 (3.2)	27.46 (3.5)	0.71
Mean systolic blood pressure (mmHg)	136.2 (14.2)	139.0 (10.4)	0.44
Mean diastolic blood pressure (mmHg)	84.0 (7.8)	88.9 (9.2)	*0.05*
Mean total Cholesterol (mg/dL)	247.5 (38.1)	251.2 (39.2)	0.74
Mean HDL (mg/dL)	56.3 (14.3)	53.5 (13.9)	0.49
Mean LDL (mg/dL)	164.3 (32.2)	171.50 (40.9)	0.49
Mean Triglycerides (mg/dL)	134.5 (57.9)	132.7 (49.5)	0.91
Mean apoA1 (mg/dL)	162.2 (39.5)	157.3 (30.6)	0.63
Mean apoB (mg/dL)	115.5 (25.1)	119.3 (22.1)	0.57
Mean glucose (mmol/mol)	89.6 (10.8)	88.1 (13.9)	0.67
Mean HbA1c (mg/dL)	35.3 (3.2)	35.9 (6.4)	0.63
Mean oxLDL (U/L)	80.8 (34.5)	69.1 (17.7)	0.16
On hypertension medication	8/26	5/24	0.42
Smoker	1/26	3/24	0.34
Mean level of perceived stress (scale 1–10)	4.9 (2.6)	5.5 (2.0)	0.44
Meat consumption > = 5x/week	8/26	7/24	0.90
Vegetarian	2/26	3/24	0.57
Daily alcohol consumption	14/26	10/24	0.39
Menopausal status in women	11/17	6/13	0.31
Mean weight	81.6 (12.8)	78.4 (10.7)	0.35

*independent samples *t*-test, chi^2^-test or Fisher’s exact test.

Values in italics are statistically significant.

Tables 3 and 4 compare the effects on biochemical parameters, cardiovascular risk markers, oxidative stress and clinical parameters in the intervention and control groups. The concentration of LDL was lowered by approximately 24% in the intervention group whereas it increased by 1% in the control group. Oxidized LDL decreased by 20% in the intervention group and increased by 5% in the control group. Total cholesterol, apoB and triglycerides decreased by 17, 6 and 16% respectively in the intervention group; in the control group they increased by 2, 6 and 16% respectively. Systolic blood pressure decreased by 10 and diastolic pressure by 7 mmHg in the intervention group. Other parameters (HDL, apoA, HbA1c, weight and waist circumference) were not altered.

**Table 3:** **Comparison between alterations in biochemical parameters in intervention and control group**

	**Intervention group**		**Placebo group**		**Difference intervention group (absolute value and %)**	**Difference placebo group (absolute value and %)**	**95% CI of difference**	**p-value**
	**Baseline intervention**		**Baseline intervention**					
LDL (SD) mg/dL	164.3 (32.2)	122.6 (19.8)	170.7 (40.9)	171.5 (41.8)	−41.7 (−23.7%) (28.3)	0.8 (0.5%) (24.3)	25.9; 56.0	*<0.001*
total CHOL (SD) mg/dL	247.5 (38.1)	204.0 (26.0)	251.2 (39.2)	255.1 (50.0)	−43.5 (−16.5%) (31.1)	4.0 (2.0%) (23.7)	31.6; 63.3	*<0.001*
HDL (SD) mg/dL	56.3 (14.3)	58.0 (13.8)	53.5 (13.9)	54.3 (14.0)	1.6 (3.5%) (4.3)	0.8 (2.4%) (7.7)	−4.3; 2.7	0.64
TG (SD) mg/dL	134.5 (57.9)	117.5 (53.9)	132.7 (49.5)	150.3 (84.4)	−16.1 (−8.7%) (37.4)	19.3 (15.9%) (64.6)	5.7; 65.1	*0.02*
apoA (SD) mg/dL	162.2 (39.5)	171.2 (5.8)	157.3 (30.6)	161.1 (31.0)	4.7 (2.9%) (9.4)	3.9 (3.1%) (18.8)	−18.5; 8.2	0.44
apoB (SD) mg/dL	115.5 (25.1)	96.6 (16.5)	119.3 (22.1)	126.3 (5.4)	−19.0 (−14.7%) (18.3)	7.0 (6.0%) (14.1)	16.6; 35.5	*<0.001*
HbA1c (SD) mmol/mol	35.3 (3.2)	36.1 (2.6)	35.9 (6.4)	36.7 (6.5)	0.9 (2.9%) (1.6)	0.7 (2.0%) (1.6)	−0.7; 1.1	0.61
oxLDL (SD) U/L	80.8 (34.5)	61.5 (21.4)	69.1 (17.7)	72.8 (22.2)	−19.4 (−20.4%) (21.7)	3.7 (5.2%) (11.3)	−35.2; −15.8	*<0.001*

Values in italics are statistically significant.

**Table 4:** **Comparison between alterations in clinical parameters in intervention and control group**

	**Intervention group**		**Placebo group**		**Diff intervention group**	**Diff placebo group**	**95% CI of difference**	**p-value**
	**Baseline intervention**		**Baseline intervention**					
Waist circumference (SD) cm	96.8 (10.2)	95.1 (15.0)	96.7 (10.4)	96.3 (10.7)	−1.7 (12.0)	0.7 (2.8)	−7.3; 2.7	0.35
Systolic blood pressure (SD) mmHg	136.2 (14.2)	125.8 (10.2)	139.0 (10.4)	138.7 (10.8)	−10.4 (11.4)	−0.3 (6.3)	−4.3; −0.23	*0.001*
Diastolic blood pressure (SD) mmHg	84.0 (7.8)	76.4 (15.4)	88.9 (9.2)	88.5 (9.1)	−7.6 (15.9)	−0.4 (5.5)	−3.4; −0.0	*0.05*
Weight (SD) kg	81.6 (12.8)	81.2 (13.5)	78.4 (10.7)	78.3 (10.1)	−0.4 (1.64)	−0.1 (2.65)	−1.5; 0.98	0.67

Values in italics are statistically significant.

In multivariate analysis, on the intervention group only, the effect on LDL, oxLDL and on blood pressure was not influenced by age, gender, or parameters of the metabolic syndrome.

### Cardiovascular risk

The cardiovascular risk according to SCORE (10-year risk of fatal CVD) ranged from 1 to 18% in the intervention group and from 1 to 46% in the control group (not normally distributed, median: 2% in both groups). The risk score changed after the intervention in 8/26 subjects in the intervention group (4 times −1%, twice −1.5%, once −4%, once −7%), mainly through the lowering of systolic blood pressure. In the control group, the risk decreased by 2% in one participant, and it increased in two people, by 1 and 3% respectively.

### Side effects

One person in the intervention group discontinued his participation after two weeks because of perceived side-effects (severe lumbal muscle ache). At the time of drop out, he reported to suffer from levo-dopa responsive dystonia (Segawa’s syndrome), a rare, autosomal dominant hereditary neurological disease. Creatine kinase (CK) was not elevated.

In the intervention group, 20/25 of the participants who completed the study reported none of the side-effects under study (see Methods section). Three participants complained of muscle ache (severity on a VAS scale 74, 37, and 16/100 respectively). One person had muscle cramps (severity: 14/100), one had arthralgia (28/100), one depression (42/100) and one sleeping problems (10/100). Other perceived side-effects in this group (each mentioned once) were hay fever, low blood pressure, constipation, ankle edema, night sweats, dry mouth.

In the control group, 21/24 participants had none of the studied side effects. Two people suffered from muscular weakness (33 and 21/100 on the VAS scale), two had arthralgia (50 and 27/100), two complained from sleeping problems (48 and 32/100), one of muscle ache (76/100), one of muscular cramps (58/100) and one of depression (27/100). One control person reported to suffer from all five of the above mentioned side-effects. Other perceived side effects were influenza, loss of muscle strength, rash, and flushes.

Mean CK level at the end of the study was 127 U/L (SD 85, range 33–376) in the intervention group and 119 U/L (SD 49, range 32–238) in the control group. Mild CK elevation (less than twice the laboratory’s cut off value of 170 in women and 190 in men) was observed in 2/24 control subjects and in 4/26 participants in the intervention group. In one patient in the intervention group this CK-elevation was accompanied by muscle ache.

## Discussion

Consumption of the food supplement under study resulted in a statistically significant lowering of total cholesterol, LDL, TG and apoB. The primary target, LDL concentration, was lowered by 24%. Oxidized LDL decreased by approximately 20% in the intervention group. The other biochemical parameters (HDL, apoA, HbA1c) were not significantly altered.

The lipid lowering effect of red yeast rice has been well documented in clinical trials [21-24]. Red yeast rice is produced by culturing the yeast *Monascus purpureus* on rice. This process results in the production of a mixture of monacolins, which are inhibitors of hydroxymethylglutaryl-coenzyme A (HMG-CoA) reductase and thus inihibit cholesterol synthesis in the liver. Monacolin K is chemically identical to the statin commercialized as lovastatin. The bioavailability of lovastatin in RYR products is higher than lovastin in tablets, which may explain why the cholesterol-lowering effect of RYR is larger than would be expected if only the dose of monacolin K is being taken into account [23].

OxLDL was lowered by one third in our intervention group. Oxidative stress is linked to the separate components of MetS [25,26]. Current evidence suggests that, as are inflammation and prothrombosis, OxLDL may be an early manifestation of MetS, rather than a consequence, and may play a central role in its development. [9,27]. Obese persons with MetS have higher oxLDL levels than obese persons without the syndrome, contributing to the greater risk of CVD disease in the MetS group [28]. In a 20-year follow up study, a higher concentration of oxidized LDL was associated with increased incidence of metabolic syndrome in the general population [29]. Therapeutic approaches which target oxidative stress may delay disease progression in persons with MetS [9]. In this context, the results of our study are promising.

Statin therapy decreases triglyceride levels in a dose dependent way, and in proportion to baseline triglyceride levels [30]. Similarly, a limited reduction in triglycerides (−9%) was observed in the intervention group in our study. Apo-B concentration is a measure for the total number of circulating atherogenic particles. Thus, lowering LDL concentration by statins is accompanied by a decrease in Apo-B, although the relationship between both levels is complex and depends on other factors such as TG concentration [31]. Similarly, Apo-B decreased by 15% in our intervention group.

Surprisingly, we observed a significant reduction of systolic as well as diastolic blood pressure in the intervention group, without significant changes in weight or waist circumference. We found no other studies with monacolins showing a reduction of blood pressure and most studies do not use blood pressure as an outcome parameter. Likewise, no rigorous RCTs on the effect of olive extract on blood pressure in humans were identified. Lowering blood pressure was one of the health claims which were rejected by EFSA for lack of solid evidence in humans. Animal studies and epidemiological studies in humans show that olive oil consumption is inversely associated with systolic and diastolic blood pressure [32,33]. The blood pressure lowering effect of olive oil (not extract) was demonstrated recently in the controlled setting of clinical trials [34,35]. The observations in this study need further exploration and confirmation.

Another issue which deserves attention is the potential diabetogenic effect of statins in a MetS population which is already prone to developing diabetes. The effect is dose-dependent and differences between statins are observed [36]. Although the benefits through LDL lowering outweigh the harms caused by the increased risk of diabetes, especially in high risk phenotypes of MetS, there is a need for novel compounds, or combination therapies, to safely lower LDL in this group [37]. In our study, we found no statistically significant increase in HbA1c levels (+2.91% vs +2.03% in the control group). Other RYR studies in the general population (not in metabolic syndrome) showed no increase in fasting glucose [38]. Whether red yeast rice extracts have a favorable profile as compared to statins in this context, remains to be studied.

Red yeast rice formulations seem to be well tolerated, even in statin-intolerant patients [24,39], but large studies to assess possible side effects have not yet been conducted. There is anecdotal evidence of side-effects similar to those of statins [40,41], and there is probably an underreporting of adverse effects.

A number of other possible problems with functional foods have been pointed out, especially the lack of regulation in the food supplement industry. Monacolin levels per gram of labeled “active product” are not standardized and there is considerable variability among products on the market [42]. It is difficult to make a comparison between formulations, and information on labels and actual content may differ. In some products, potentially harmful levels of suspected nephrotoxins have been found [42]. Monacolins are a class of compounds produced during fermentation, and the composition can be influenced by the fermentation conditions. Furthermore the composition of Monacolins can be changed due to stability issues (degradation) while very high levels, i.e. too high to be the result of a natural fermentation process, might suggest that the product has been spiked with chemical lovastatin [43,44]. Standardized production and batch-to-batch control are needed.

The short term follow up period of 10 weeks is a limitation of this study. Furthermore, the sample size of this study was sufficient to examine the effectiveness of the study product, but does not allow to adequately assess side-effects. Thirdly, in this study design it was not possible to assess to which component of the product under study (red yeast rice or olive extract) the observed effects can be attributed, and whether or not there is an additional, synergistic or even antagonistic effect of combining both components.

## Conclusions

In this double blind, placebo controlled randomized trial in people with metabolic syndrome, daily consumption of a food supplement combining RYR and olive fruit extract resulted in a 24% decrease of LDL concentrations, accompanied by a smaller but significant decrease of total CHOL, apoB, and TG. Oxidized LDL was decreased by 20%. Both systolic and diastolic blood pressure decreased by 10 and 7 mmHg respectively. The combination of extracts in this study may be an alternative approach to statins in people who cannot or do not want to be treated with statins. The clinical use of this class of products should be restricted to formulations which have been studied and characterized according to current research standards, and patients need to be informed about the possibility of adverse effects.
